# ﻿Topotypes of the millipede species *Kronopolitesswinhoei* (Pocock, 1895) reveal a new synonym with revalidation of *Kronopolitessvenhedini* (Verhoeff, 1934) (Diplopoda, Polydesmida, Paradoxosomatidae)

**DOI:** 10.3897/zookeys.1231.137769

**Published:** 2025-03-10

**Authors:** Yuan Xiong, Huiming Chen, Xuankong Jiang, Chao Jiang

**Affiliations:** 1 State Key Laboratory for Quality Ensurance and Sustainable Use of Dao-di Herbs, National Resource Center for Chinese Materia Medica, China Academy of Chinese Medical Sciences, 100700, Beijing, China China Academy of Chinese Medical Sciences Beijing China; 2 Institute of Biology, Guizhou Academy of Sciences, Guiyang 550009, China Guizhou Academy of Sciences Guiyang China

**Keywords:** New combination, revalidation, revision, taxonomy, topotypes

## Abstract

The millipede genus *Kronopolites* Attems, 1914 was originally described by monotypy with *Strongylosomaswinhoei* Pocock, 1895 as the type species, which was based on a single female specimen. Although this species has been believed to be widespread in China, there have been no confirmed reports of it from its type locality, leading to uncertainty about its taxonomic status. To address this issue, we newly sampled specimens from its type locality in Zhifu, Shandong Province, China. Our morphological analysis suggests that *Kronopolitesswinhoei* (Pocock, 1895) should be reclassified as *Nedyopusswinhoei* (Pocock, 1895) **comb. nov.** and is a senior synonym of *Nedyopuspatrioticus* Attems, 1898, **syn. nov.** The results also support the recovery of the name *Kronopolitessvenhedini* (Verhoeff, 1934) **sp. reval.**, which was previously misidentified as a junior synonym under *K.swinhoei*. The former is now the genus type of *Kronopolites*.

## ﻿Introduction

Pocock (1895) described Chilopoda and Diplopoda from the coastal regions of China and Japan and treated 18 millipede species, including 17 species new to science. These are some of the earliest millipede findings documented in China. *Strongylosomaswinhoei* Pocock, 1895 was initially described based on exclusively a female specimen from Zhifu (= Chee Foo) Island in Yantai, Shandong Province. In 1914, Attems established the genus *Kronopolites* by monotypy with this species. However, subsequent identification by Brölemann (1896) of specimens from Zhoushan Island, Zhejiang Province as *K.swinhoei*, without examination of the gonopods, led to the premature acceptance of its wider distribution. To date, *K.swinhoei* has been documented in Chongqing, Gansu, Guizhou, Qinghai, Shaanxi, Shandong, Sichuan, Yunnan, and Zhejiang provinces (Golovatch 2019; Chen et al. 2023); all of these are geographically distant from Zhifu Island, Shandong Province. This casted further doubt on the validity of the identification of this species.

Attems (1914) established the genus *Kronopolites* by monotypy with *Strongylosomaswinhoei* (Pocock, 1895), based on the wide tibia of the gonopod. The genus *Kronopolites* has since undergone two revisions, first by Hoffman (1962) and later by Likhitrakarn et al. (2015), and currently comprises 12 species, which are mainly distributed in China, Kashmir Himalaya, Laos, Nepal, Thailand, and Vietnam, with five species found in China (Golovatch 2020; Golovatch and Semenyuk 2021). However, the male of *K.swinhoei* has never been verified from its type locality, which poses challenges to the comprehensive understanding of this species and the genus (Hoffman 1962).

To address this issue, millipedes closely matching Pocock’s description were collected on Zhifu Island and identified as topotypic *K.swinhoei*. Subsequent morphological studies indicate that Brölemann’s identification of *K.swinhoei* was incorrect. Consequently, it is determined that *K.swinhoei* belongs to the genus *Nedyopus* and is a senior synonym of *Nedyopuspatrioticus* (Attems, 1898), which is widely distributed in East Asia. This removal of *K.swinhoei* from *Kronopolites* leads to the revalidation and reassignment of *Kronopolitessvenhedini* (Verhoeff, 1934), instead, as the type species of the genus *Kronopolites*.

## ﻿Materials and methods

Specimens were collected by tweezers and preserved in 75% ethanol for morphological studies. Live animals were photographed with a Sony A7R4A camera with a Sony FE 90 mm macro lens. Specimens are deposited in National Resource Center for Chinese Materia Medica, China Academy of Chinese Medical Sciences, Beijing, China (**CMMI**) and the Institute of Biology, Guizhou Academy of Sciences, Guiyang, China (**IBGAS**). All specimens deposited in CMMI are numbered and stored according to the collection date (year/month/day) plus an auto-incrementing serial number. If the dates are the same for the same collection site, only the numbers will be retained (e.g. 20230922044 and 20230922045 will be changed into 20230922044 and -45).

Specimens were examined, photographed, and measured using a Leica M205 MCA microscope equipped with a Leica DMC 6200 camera and LAS software v. 4.1 (Leica, Germany). Photos were converted into hand-drawn illustrations using SKETCHBOOK v. 6.0.6. Maps were generated with ArcMap v. 10.7.1 software (Figs 4, 7). Grammarly was used to polish English in the manuscript. Terminology is from Hoffman (1962), Chen et al. (2006b), and Likhitrakarn et al. (2015).

## ﻿Results

### ﻿Taxonomy

**Family Paradoxosomatidae Daday, 1889**

**Subfamily Paradoxosomatinae Daday, 1889**

#### ﻿Tribe Nedyopodini Attems, 1898

##### ﻿Genus Nedyopus

Taxon classificationAnimaliaPolydesmidaParadoxosomatidae

Attems, 1914

AACC699E-8918-5D8A-A0C1-7DB1825B24F7

###### Type species.

*Orthomorphacingulata* Attems, 1898, by original designation.

##### ﻿ Nedyopus swinhoei

Taxon classificationAnimaliaPolydesmidaParadoxosomatidae

(Pocock, 1895) comb. nov.

8C1324A3-D28A-523B-969C-713702653B4A

Figs 1A , 2 , 3 , 4

*StronglosomaSwinhoei* Pocock, 1895: 354–355. Type specimen: holotype female, collected from Chee Foo (= Zhifu), Yantai, Shandong Province of China, deposited at the British Museum of Natural History (Hoffman 1962), not examined.
Kronopolites swinhoei : Attems 1914.
Strongylosoma patrioticum Attems, 1898: 300, figs 12, 13. Type locality: Japan. New synonymy.
Nedyopus patrioticus : Attems 1914: 201; Attems 1937: 138–139; Takashima 1949:17; Takakuwa 1954: 47; Takashima and Haga 1956: 332; Miyosi 1959: 49, 71; Wang 1964: 71; Wang 1996: 87; Mikhaljova et al. 2000: 119; Korsós 2004: 23; Chen et al. 2006b: 3998–4000; Nguyen and Sierwald 2013: 1231. New synonymy.

###### Diagnosis.

Differs from other species of the genus by the following combination of characters. The metaterga have strong contrasting colors, which are not circularly patterned as in other *Nedyopus* species, and the gonopod femur suddenly widens at the base, with l’ and l” not jagged.

###### Material examined.

**China** – **Anhui Province**: • 1 ♂, Fuyang, 30.I.2020, Yihao Ge leg. (IBGAS). • **Jiangxi Province**: 1 ♂ and 1 ♀, Ji’an, (27.1439°N, 115.0426°E), 50 m a.s.l., 19.V.2017, Xuankong Jiang leg. (IBGAS). • **Shandong Province**: 1 ♂ and 1 ♀ (20231025001), Yantai, Zhifu District, Zhifu Island, (37.6100°N, 121.3716°E), 40 m a.s.l., 25.X.2023, Xuankong Jiang, Tian Lu and Chongwu Lu leg. (CMMI); • 16 ♂♂ and 12 ♀♀, same data, (IBGAS);• 15 ♂♂ and 13 ♀♀, Yantai, Zhifu District, Tashan Park, (37.5056°N, 121.3972°E), 290 m a.s.l., 24.X.2023, Xuankong Jiang, Tian Lu and Chongwu Lu leg. (IBGAS).

###### Description.

**Figure 1.**
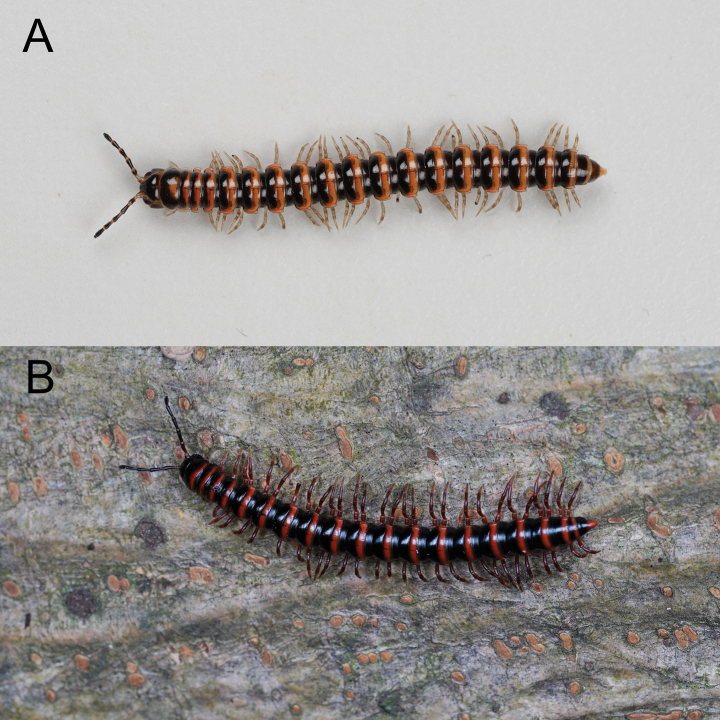
Live specimens **A***Nedyopusswinhoei* (Pocock, 1895) comb. nov. from Zhifu Island, Shandong, China **B***Kronopolitessvenhedini* (Verhoeff, 1934) sp. reval. from Xinyang, Henan, China. Not to scale. Photographs by Xuankong Jiang.

**Figure 2.**
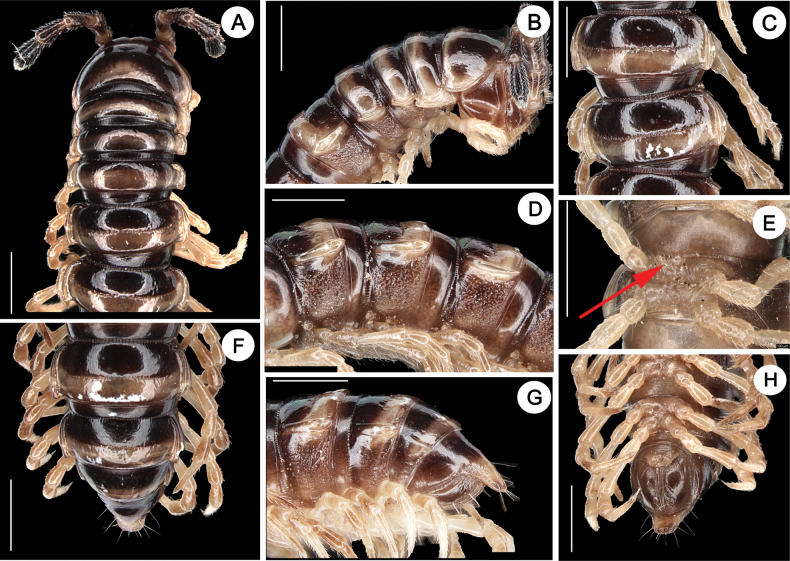
*Nedyopusswinhoei* comb. nov. **A** anterior part of body, dorsal view **B** anterior part of body, lateral view **C** segments 10 and 11, dorsal view **D** segments 9–11, lateral view **E** sternal cones between coxae 4, anterior view **F–H** posterior part of body, dorsal, lateral, and ventral view, respectively. Scale bar: 1 mm.

Length ca 17.5–25.1 mm (♂), 18.2–32.7 mm (♀) with 20 segments. Live color variable (Fig. 1A). Posterior half of each metazonae with transverse band, pale yellow to orange. Antennomere 1–6 dark brown, antennomere 7 whitish. Legs light yellow (Fig. 2A–H).

Clypeolabral region and vertex densely setose. Epicranial suture distinct. Width of body gradually expanded from head to 5^th^ segment, approximately equal in width from 5^th^ to 16^th^ segments, and tapering from 16^th^ to telson. Caudal corner of collum broadly rounded, declined ventrad, produced behind rear tergal margin (Fig. 2A, B).

Cuticle shining (Fig. 2A, C, F); surface below paranota finely microgranulate (Fig. 2B, D, G). Paranota strongly developed (Fig. 2A, B, C, F), slightly upturned, lying rather high (at upper 1/3 of body) but below dorsum; anterior edge broadly rounded and narrowly bordered; posterior edge nearly straight. Ozopores evident, lying on paranota at its posterior margin, in segments 5, 7, 9, 10, 12, 13, 15–19. Transverse sulcus usually distinct (Fig. 2A, C, F), complete on metaterga 5–18 (♂), narrow, linear, shallow, reaching bases of paranota, faintly ribbed at bottom. Stricture between pro- and metazonae evident, broad and deep, ribbed at dorsal side down to base of paranota (Fig. 2A–F). Pleurosternal carinae with a sharp caudal tooth on segments 3–6. Epiproct (Fig. 2G, H) conical, flattened dorsoventrally; tip subtruncate; pre-apical papillae small, lying close to tip. Hypoproct roundly subtriangular, spinnerets at caudal edge small and well separated (Fig. 2H). Sterna densely setose, without modifications, but with two small, rounded, fully separated, setose cones between ♂ coxae 4 (Fig. 2E).

**Figure 3.**
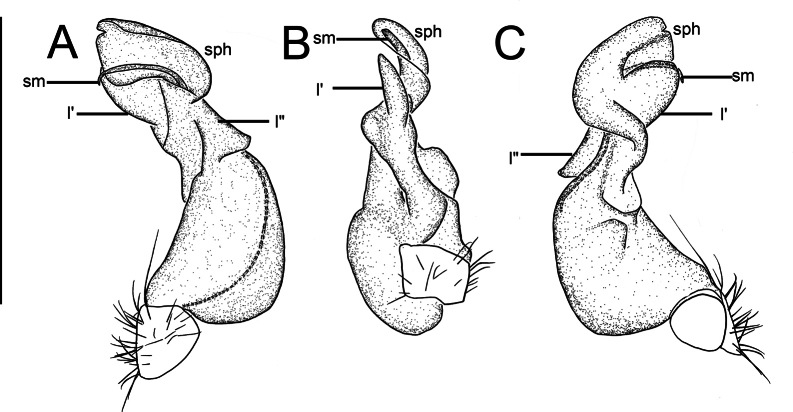
*Nedyopusswinhoei* comb. nov., left gonopod **A** lateral view **B** dorsal view **C** mesal view. sm, solenomere; sph, solenophore; l’, l”. Scale bar: 1 mm.

Gonopods (Fig. 3) intricate. Coxite elongate, subcylindrical, strongly setose distoventrally. Prefemoral part nearly half femoral length. Femorite short and bulge out at one end like a belly, distal portion carrying two lobes (l’ and l”). l’ parallel to solenophore. Solenophore lamelliform, twisted distally. Solenomere short and flagelliform.

###### Distribution.

China: Anhui (New record), Jiangsu, Jiangxi (New record), Shandong, Taiwan (Pocock 1895; Chen et al. 2006b; Zhang et al. 2024); Indonesia, Japan, Korea (Nguyen and Sierwald 2013).

###### Remarks.

The specimens from Zhoushan Island were initially identified as *K.swinhoei* by Brölemann in 1896, without providing a justification. However, our investigation reveals a distinct divergence from the original description. For instance, the specimens from Zhoushan are notably larger (47 mm vs 35 mm) and have more vivid in color on the metazonites (orange-red vs yellow).

During our research in Zhifu, we found a species that closely matches Pocock’s description, leading us to confidently identify it as *K.swinhoei*. On examination of the topotypes, we observed significant differences in the gonopods compared to Brölemann’s illustrations (1896). These differences, including the femorite (strongly twisted and expanded in *Nedyopus* vs straight in *Kronopolites*), the postfemoral sulcus (missing in *Nedyopus* vs existed in *Kronopolites*) and the solenophore (lamelliform in *Nedyopus* vs tubuliform in *Kronopolites*), indicate that this species belongs to *Nedyopus* rather than *Kronopolites*, and is identical to the widespread species *Nedyopuspatrioticus* (Attems, 1898). Consequently, *K.swinhoei* is formally transferred to *Nedyopus*, and *Nedyopuspatrioticus* is considered a junior synonym of *Nedyopusswinhoei* (Pocock, 1895) comb. nov. Additionally, *N.patrioticus* consists of two subspecies *Nedyopuspatrioticuspatrioticus* (Attems, 1898) from Japan and *Nedyopuspatrioticusunicolor* (Carl, 1902) from Indonesia. Therefore, the subspecies *unicolor* should be treated as *Nedyopusswinhoeiunicolor* (Carl, 1902) comb. nov.

**Figure 4.**
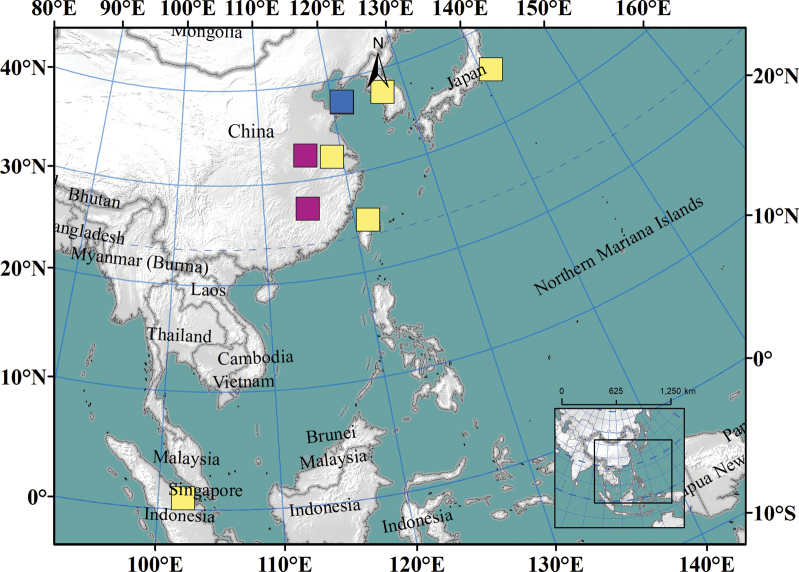
Distribution of *Nedyopusswinhoei* comb. nov. Yellow: literature records; purple: specimens collected in this article; blue: type locality.

*Nedyopusswinhoei* (Pocock, 1895) comb. nov. has a wide distribution across Asia, from Indonesia to China, Korea, and Japan (Wang 1955; Nguyen and Sierwald 2013). This species is often found near human habitation and may spread through human activities. In China, documented records of this species were limited to Jiangsu, Shandong, and Taiwan provinces (Pocock 1895; Wang 1955; Zhang et al. 2024), possibly due to limited investigation. Our study identified additional distribution locations in Anhui and Jiangxi, suggesting that the species might be found in other regions across China in the future.

#### ﻿Tribe Sulciferini Attems, 1898

##### ﻿Genus Kronopolites

Taxon classificationAnimaliaPolydesmidaParadoxosomatidae

Attems, 1914

5AB21EA3-7021-59E1-AE10-A74965A15120

Kronopolites Attems 1914: 219.
Kronopolites : Attems 1929: 272; 1931: 113; 1936: 225; 1937: 49; Verhoeff 1939: 274; Takashima 1950: 38; Takakuwa 1954: 30; Hoffman 1962: 579; 1980: 169; Jeekel 1971: 225; 1982: 243; 1988: 98; Chen et al. 2006a: 252; Golovatch 2009: 121; 2013: 12; Nguyen and Sierwald 2013: 1286; Likhitrakarn et al. 2015: 32; Golovatch 2015: 135; Golovatch 2016: 1; Golovatch 2019: 348; Golovatch and Liu 2020: 170; Golovatch and Semenyuk 2021: 479.
Kansupus Verhoeff 1934: 17, synonymized by Attems (1936: 233).
Kansupus : Jeekel 1971: 225; Hoffman 1980: 169.
Parakansupus Verhoeff 1939: 273, synonymized by Hoffman (1962: 579).
Parakansupus : Jeekel 1971: 230; Hoffman 1980: 169.

###### Type species.

*Kronopolitessvenhedini* (Verhoeff, 1934) sp. reval., by present designation.

###### Diagnosis.

See Likhitrakarn et al. (2015: 29).

###### Remarks.

Attems (1914) established the genus and designated *Strongylosomaswinhoei* Pocock, 1895 as the type species by monotypy. However, due to the misidentification of this species, which was later transferred to *Nedyopus*, the correct designation by Attems, *Kronopolitessvenhedini* (Verhoeff, 1934) sp. reval., has now been reclassified as the type species of the genus.

##### ﻿ Kronopolites svenhedini

Taxon classificationAnimaliaPolydesmidaParadoxosomatidae

(Verhoeff, 1934) sp. reval.

1B4CA5D2-D003-5ACE-B00D-F66E0D1C04E9

Figs 1B , 5 , 6 , 7

*StronglosomaSwinhoei*: Brölemann 1896: 354–357, fig. 9–11; Attems 1898: 304 (misidentified).
Kansupus svenhedini Verhoeff, 1934: 17, figs.4–8, synonymized by Hoffman 1962: 581. Type locality: N. O. Szetschuan (= northeastern Sichuan Province), China; ♂ and Süd-Kansu (= Longnan, Gansu Province, China; ♀).
Kronopolites swinhoei : Attems 1914: 219; Attems 1936: 226, fig. 44; Attems 1937: 50–51, fig. 64; Chamberlin and Wang 1953: 5; Hoffman 1962: 581–583, figs 1, 2; Golovatch 1978: 678; 1982: 298; Wang 1996: 86; Geoffroy and Golovatch 2004: 20; Korsós 2004: 23; Chen et al. 2006a: 252; Golovatch 2009: 121; 2013: 2, figs 1–4; Nguyen and Sierwald 2013: 1286; Likhitrakarn et al. 2015: 32; Golovatch 2016: 1; 2019: 348, figs 4, 5; 2020: 166; Golovatch and Liu 2020: 170; Golovatch and Semenyuk 2021: 479, figs 28–30 (misidentified).
Kronopolites svenhedini : Attems 1936: 233; 1937: 53, fig. 66; Zhang and Li 1978: 12; Wang 1996: 86.
Kansupus svenhedini var. dentiger Verhoeff, 1934: 19, fig. 9; Attems 1936: 233; Attems 1937: 54, synonymized with Kronopolitesswinhoei by Hoffman 1962: 581. Type locality: Pei-shui-ho (= Baishuijiang National Nature Reserve), Wen County, Gansu Province, China).

###### Material examined.

**China** – • **Gansu Province**: 5 ♂♂ and 40 ♀♀ (20230922044, -45, -46, 20230922048–20230922051, 20230922054–20230922057, -60, -61, -63, -66, -67, -68, -70, -72, -73, -75, -76, 20230922078–20230922090, 20230922092–20230922098, -101, -102, -104), Lintao County, Fenghuangshan Forest Park (35.4009°N, 103.8901°E), 1960 m a.s.l., 22.IX.2023, Tianyun Chen, Jiabo Fan & Yiying Zhao leg., (CMMI); • 2 ♂♂ and 2 ♀♀, Dingxi City, Anding District, Guanying Town, Yawan Village, 27.VI.2008, Zhiyong Di leg., (IBGAS). • **Henan Province**: 1 ♂ and 2 ♀♀, Xinyang City, Dabieshan station (32.1252°N, 114.0118°E), 110 m a.s.l., 10.VIII.2023, Xuankong Jiang & Leilei Shi leg., (IBGAS);• 1 ♂ and 1 ♀ 5J, Xinyang City, Shihe district, Bailongtan Reservoir (31.9987°N, 113.9105°E), 230 m a.s.l., 10.VIII.2023, Xuankong Jiang & Leilei Shi leg., (IBGAS); • 17 ♂♂ and 5 ♀♀ 3J, Xinyang, Nanwan Reservoir (32.1252°N, 114.0118°E), 110 m a.s.l., 9.VIII.2023, Xuankong Jiang & Leilei Shi leg., (IBGAS). • **Qinghai Province**: 3 ♂♂ and 10 ♀♀, Huangnan Tibetan Autonomous Prefecture, Jianzha County, Zhiyong Di leg., (IBGAS). • **Shaanxi Province**: 1 ♂ (20190907029), Mei County, Honghegu National Forest Park (34.0533°N, 107.7836°E), 1730 m a.s.l., 7.IX.2019, Chao Jiang leg., (CMMI); • 3 ♂♂ and 4 ♀♀ (20200905117, -118, 20230802001, -02), Xi’an, Huyi District, Taiping National Forest Park (33.9951°N, 108.7163°E), 540 m a.s.l., 2.VIII.2023, Tianyun Chen &Yuan Xiong leg., (CMMI). • **Zhejiang Province**: 3 ♂♂ and 3 ♀♀, Anji County, Longwangshan Scenic Area, 22.VII.2018, Rong Fu leg., (IBGAS).

###### Diagnosis.

Differs from other species of the genus by the following combination of characters: metazonae have two shapes, either as a transverse band or a median oval spot, and also have two color variations, ranging from pale yellow to orange-red; paraterga relatively poorly developed, set lower (mostly at about 1/3 height of segments), caudal corners usually not surpassing rear tergal contours, at most narrowly rounded; ♂ sternal cones present; processes *a* and *b* of gonopod on a broad common stem, neither slender nor long (Golovatch 2009).

###### Description.

**Figure 5.**
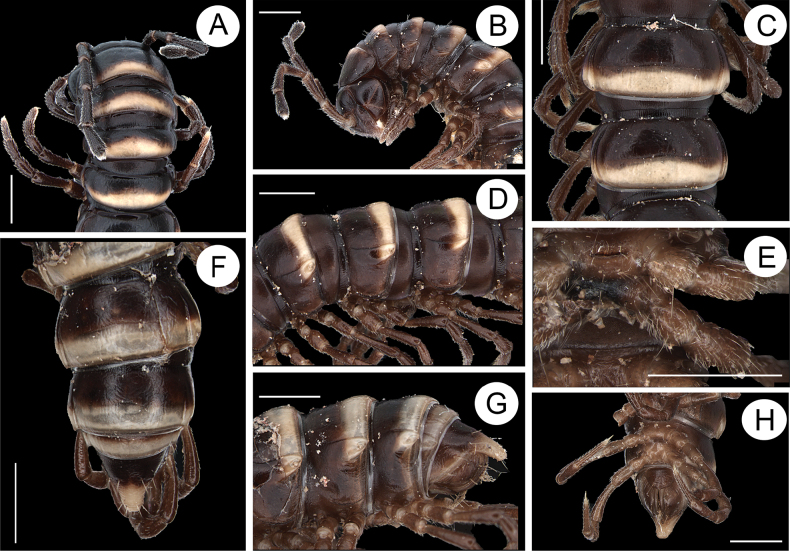
*Kronopolitessvenhedini* sp. reval **A** anterior part of body, dorsal view **B** anterior part of body, lateral view **C** segments 10 and 11, dorsal view **D** segments 9–11, lateral view **E** sternal cones between coxae 4, ventral view **F–H** posterior part of body, dorsal, lateral and ventral views, respectively. Scale bars: 1 mm.

Length ca 26.0–50.0 mm (♂), 27.0–60.0 mm (♀) with 20 segments. Live color variable (Fig. 1B). Head, prozonae, anterior part of collum, and metazonae black; posterior part of collum and each metazonite with transverse band or oval spot, pale yellow to orange-red. If the patches not covering ozopores, then the ozopores exhibit the same color of the patches. Telson black with color of tip identical to the patches. Antennae black; legs black to reddish brown (Fig. 5A–H).

Head densely setose. Antennae moderately long (Fig. 5A), extending behind body segment 3 when stretched dorsally. Width gradually widened from collum to 5^th^ segment, roughly equal in 5^th^–16^th^ segments, and tapering from 16^th^ to telson.

Collum of different specimens with one or two transverse rows of setae, one row with 1+1 anterior, two rows with 1+1 at both anterior and intermediate. Caudal corner of collum very broadly rounded, declined ventrad, produced behind rear tergal margin (Fig. 5A, B).

Cuticle shining, prozonae finely shagreened, metaterga finely rugulose (Fig. 5A, C, F), surface below paranota finely microgranulate (Fig. 5B, D). Postcollum metaterga with one transverse row of setae: 4+4 or 3+3 in anterior (pre-sulcus). Tergal setae long and slender, mostly abraded. Paranota well developed (Fig. 5A–F), lying rather high (at upper 1/2 of body), arched. Ozopores evident, lateral, lying in an ovoid groove at about 1/4 in front of posterior edge of metaterga, in segments 5, 7, 9, 10, 12, 13, 15–19. Transverse sulcus usually distinct (Fig. 5A, C, F), slightly incomplete on segment 18, complete on metaterga 3–18 (♂), narrow, linear, shallow, reaching bases of paranota, faintly ribbed at bottom. Stricture between pro- and metazonae evident, broad and deep, ribbed at bottom down to base of paranota (Fig. 5A, B, D, G). Pleurosternal carinae with a sharp caudal tooth on segments 2–7, thereafter increasingly strongly reduced until 18^th^ segment (♂). Epiproct (Fig. 5G, H) conical, flattened dorsoventrally, with two small apical papillae; tip subtruncate; pre-apical papillae small, lying close to tip. Hypoproct roundly subtriangular, spinnerets at caudal edge small and well separated (Fig. 5H). Sterna densely setose with a long tongue-shaped process on ♂ coxae 4 (Fig. 5E). Legs rather long and slender (Fig. 5A, B, D, G).

**Figure 6.**
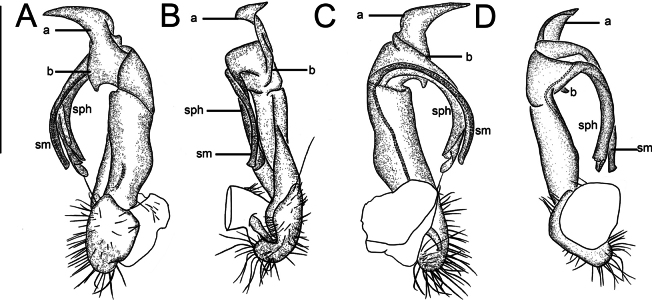
*Kronopolitessvenhedini* sp. reval, left gonopod **A** ventral view **B** mesal view **C** dorsal view **D** lateral view. sm, solenomere; sph, solenophore; a, b. Scale bar: 1 mm.

Coxite of gonopods (Fig. 6) thick, pressing inwards on the spermathecal fossa in the prefemur. Prefemur short, with numerous slender setae. Femorite rather stout, with an evident mesal groove and a strong distolateral sulcus demarcating a postfemoral part; the latter well developed, with very prominent bipartite, crescent-shaped, lateral processes: process *a* rather long and broad; process *b* short, broad and pointed. Solenophore in the form of a long, tubular branch. Solenomere flagelliform.

###### Distribution.

China: Chongqing, Qinghai, Gansu, Guizhou, Henan (new record), Shaanxi, Sichuan, Yunnan and Zhejiang (Golovatch 2019; Chen et al. 2023).

###### Remarks.

Verhoeff (1934) described a new monotypic genus and species, *Kansupussvenhedini*, based on specimens from Gansu, China. Later, Attems (1936) and Hoffman (1962) synonymized the name with *Kronopolites* and *K.swinhoei* respectively, relying on Brölemann’s (1896) description. However, our research shows that Brölemann (1896) misidentified this species. Thus, *K.svenhedini* sp. reval. should be resurrected. To maintain the stability of the taxonomic name *Kronopolites*, the type species of *Kronopolites* now fixed (under Article 70.3 of the International Code of Zoological Nomenclature 1999) as *Kronopolitessvenhedini* (Verhoeff, 1934), which was misidentified as *Kronopolitesswinhoei* (Pocock, 1895) in the original designation by Attems (1914). Furthermore, the publications mentioning this species did not state where the type specimen is preserved, rendering the location of its storage unknown (Verhoeff 1934; Attems 1936, 1937; Hoffman 1962; Zhang and Li 1978; Wang 1996).

**Figure 7.**
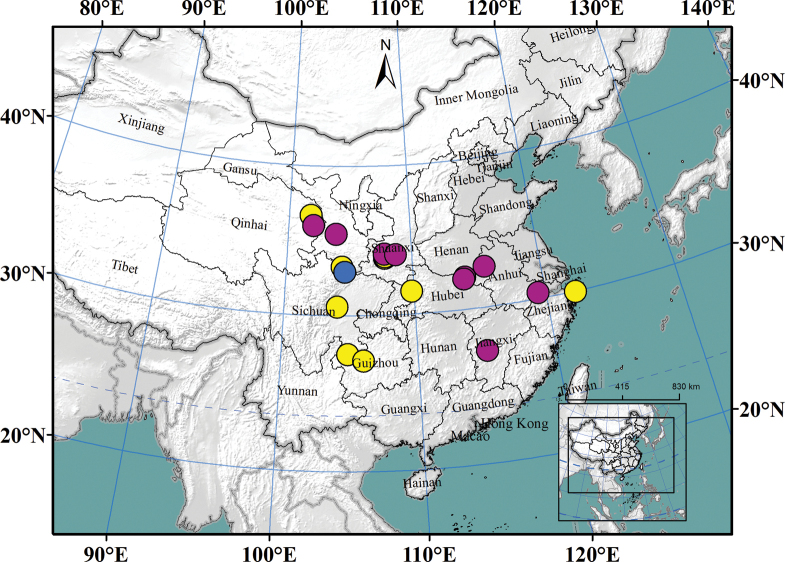
Distribution of *Kronopolitessvenhedini* sp. reval. Yellow: literature records; purple: specimens collected in this article. blue: type locality.

*Kronopolitessvenhedini* (Verhoeff, 1934) sp. reval. is widely distributed in China, with the westernmost occurrence in Qinghai, the easternmost in Zhejiang, the southernmost in Yunnan and the northernmost in Gansu. It shows variation in color, body size, and subtle differences in gonopod shape among different populations (Golovatch 2013). These differences may be attributed to geographic isolation or varying habitats, suggesting the potential presence of cryptic species. This hypothesis could be explored in future research using molecular methods.

## Supplementary Material

XML Treatment for Nedyopus

XML Treatment for Nedyopus swinhoei

XML Treatment for Kronopolites

XML Treatment for Kronopolites svenhedini

## ﻿Additional information

### Author ORCIDs

Yuan Xiong https://orcid.org/0009-0000-3812-9932

Huiming Chen https://orcid.org/0000-0002-2449-3036

Xuankong Jiang https://orcid.org/0000-0003-3506-5894

Chao Jiang https://orcid.org/0000-0003-1841-1169

### Data availability

All of the data that support the findings of this study are available in the main text.
